# Crystalline Lens Shape During Accommodation in Children

**DOI:** 10.1007/s44402-026-00069-5

**Published:** 2026-04-17

**Authors:** Asik Pradhan, Rohan P. J. Hughes, Emily C. Woodman-Pieterse, Stephen J. Vincent, Scott A. Read, David A. Atchison, Andrew Carkeet

**Affiliations:** Centre for Vision and Eye Research, Optometry and Vision Science, Queensland University of Technology, Brisbane, Queensland Australia

**Keywords:** Accommodation, Astigmatism, IOLMaster 700, Lens shape, Myopia

## Abstract

**Purpose:**

To determine how accommodation affects lens shape in myopic and non-myopic children.

**Methods:**

Participants included 76 non-myopic (spherical equivalent refraction (SER): 0.00 to +1.75 D) and 18 myopic children (SER = −3.50 to −0.75 D) aged 5−12 years. Anterior and posterior lens surface shapes were determined by image processing and ray tracing of IOLMaster 700-generated B-scan images (six meridians per scan) at 0, 3, 6 and 9 D accommodation demands, expressed as refractive power vectors (*M, J*_0_ and *J*_45_).

**Results:**

For all children, anterior and posterior lens surface *M* and total lens power increased by means (±SD) of +0.45 ± 0.17, +0.23 ± 0.20 and +0.65 ± 0.32 D per dioptre of accommodation demand, respectively, with a shift towards a more equiconvex shape with increasing accommodation. *J*_0_ and *J*_45_ did not change significantly during accommodation (for anterior surface, *p* = 0.68 and 0.48, respectively; for posterior surface, *p* = 0.47 and 0.88, respectively). Myopic lenses had significantly lower anterior and posterior surface *M* and total lens power than non-myopic lenses (*p* < 0.05), but no significant differences were observed in astigmatic vectors except for anterior lens surface *J*_0_. There were no interactions between accommodation demand and refractive error for any lens parameters (all *p* ≥ 0.13).

**Conclusion:**

Myopic children have flatter lens surfaces than non-myopic children. Lens surface shape changes with accommodation are similar between myopic and non-myopic children.

Key points

- Myopes have flatter lens surfaces and lower equivalent lens power than non-myopes.
- Accommodation-induced lens surface shape changes are similar between myopic and non-myopic children.
- Lens surface astigmatism (*J*_0_ and *J*_45_) for both lenticular surfaces does not change with accommodation.

## Introduction

The role of near work in the onset and progression of myopia has been extensively investigated in children and adults [1]. During near work, the eyes accommodate, converge and the pupils constrict to facilitate clear and single vision. Accommodation is a potential underlying mechanism that may link myopia and near work [2, 3].

During accommodation, the crystalline lens curvature, position, thickness and diameter change to vary the dioptric power of the eye, in order to facilitate clear vision over a range of viewing distances [4–9]. These lens shape changes, along with changes in anterior and vitreous chamber depth, contribute to a myopic shift during accommodation [4, 5, 10]. These changes are approximately proportional to accommodation demand and are observed in both non-myopic and myopic children [11] and young adults [12]. Furthermore, an increase in with-the-rule (WTR) astigmatism has been observed during accommodation in emmetropic [13–15] and ametropic [16–18] children and adults. Children possess higher accommodative amplitudes than adults and typically maintain closer near working distances [19–21]. Shorter working distances (<30 cm) and continuous periods of uninterrupted near work (>30 min) are reported to increase the likelihood of developing myopia in school children [19, 22, 23].

During relaxed accommodation, myopic eyes have thinner and flatter crystalline lenses than non-myopic eyes, resulting in a lower equivalent lens power [24–28]. Established myopes exhibit higher accommodative lags [29–31], which some authors have suggested may be a mechanism linking near work and myopia development and progression [29, 32–34], although others believe that higher lags are a consequence rather than a cause of myopia [35–37]. Two previous studies have found no significant difference in lens thickness change during accommodation between myopes and non-myopes [11, 12]; however, comparisons in lens surface shape between myopes and non-myopes during accommodation are limited.

Obtaining in vivo crystalline lens data is challenging because lens shape measurements are indirect, being affected by the media anterior to the lens. In addition, accommodation-induced pupillary constriction can complicate accommodative measurements. Previous studies have assessed lens shape changes in adults using Purkinje imaging [4, 38], magnetic resonance imaging (MRI) [39, 40], Scheimpflug imaging [38, 41] and optical coherence tomography (OCT) [6, 42–45]. These studies have been limited by assuming rotationally symmetrical surfaces without quantifying surface astigmatism [4, 38, 41–46].

Since crystalline lens shape and power may play roles in myopia onset and development, a novel technique [47] was used in this study to determine lens surface power and toricity from IOLMaster 700 OCT images obtained during accommodation in myopic and non-myopic children.

## Methods

This study analysed B-scan images collected as part of a project assessing ocular biometry in children during accommodation [10, 11]. Ethics approval was obtained from the Queensland University of Technology Human Research Ethics Committee.

Ocular biometry was obtained using the IOLMaster 700 (Carl Zeiss Meditec AG, zeiss.com) from 94 children (55% male, 45% female) aged 5–12 years who were classified based on non-cycloplegic spherical equivalent refraction (SER) as either non-myopes (SER > –0.50 DS) or myopes (≤–0.50 DS). All children had astigmatism ≤0.75 DC and anisometropia ≤1.00 DS. Data from 76 non-myopic children (mean age 8.5 ± 1.6 years, range 5–12 years) and 18 myopic children (mean age 10.1 ± 1.4 years, range 7.3–12.7 years) were included. Mean SERs were +0.62 ± 0.35 D (range 0.00–+1.75 D) and –2.08 ± 0.92 D (range –0.75 to –3.50 D) for the non-myopes and myopes, respectively.

Some participant data were excluded because the OCT image quality was too poor to analyse. Thus, the sample size varied with accommodation demand and refractive group. Furthermore, per the methodology outlined by Hughes et al. [10, 11], participant data were included only if they exhibited active accommodation, defined as a measurable decrease in anterior chamber depth (ACD) (<0 µm) and an increase in lens thickness (LT) (>0 µm), for a given accommodation demand. The final sample sizes for the non-myopic participants (*n* = 76) were 73, 58, 64 and 57 for the 0, 3, 6 and 9 D accommodation demands, respectively. For the myopic group (*n* = 18), 18, 16, 16 and 10 participants were analysed for the 0, 3, 6 and 9 D accommodation demands, respectively.

### Lens Shape

**Fig. 1:**
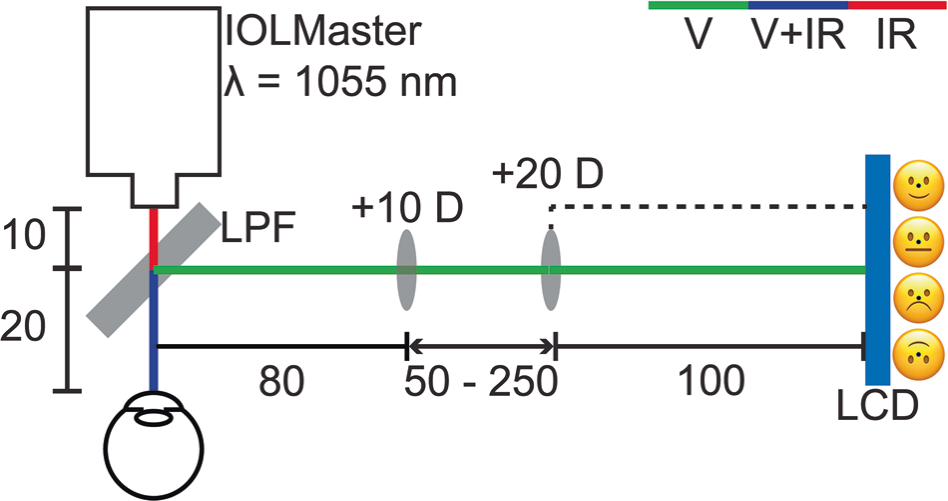
Badal system with integrated emoticon fixation targets. All distances are millimetres. IR: infra-red (>650 nm); V: visible light (400–650 nm); LPF: long pass dichroic filter; LCD: liquid crystal display. Figure reproduced from Hughes et al. [10] with permission from Wiley and Sons.

Lens shape was based on biometric measurements and meridional OCT scans from the left eyes of participants using the IOLMaster 700. Accommodation demands were presented in randomised order using a Badal system attached to the instrument (Fig. 1) [10, 11]. All participants were corrected for their SER using the Badal system during measurements. This ensured an accurate assessment of the accommodation response to the targets presented at various accommodation demands.

B-scan images were captured at six meridians (180°, 30°, 60°, 90°, 120° and 150°) using the IOLMaster and analysed as described previously [47]. Briefly, the images were corrected for distortions, the anterior and posterior lens surfaces were segmented and ray tracing was used to determine the surface radii of curvatures. Detailed quantification methods and the equations used for data analysis are provided in Supplementary file 1.

Lens surface refractive power vectors [48] *M, J*_0_ and *J*_45_, and the equivalent lens mean spherical power (*F*_L_) were determined at each accommodation demand using refractive indices of 1.376 for the cornea and 1.336 for the aqueous and vitreous humour (see Supplementary file 1). For the lens, equivalent refractive index based on age was calculated using the equation of Mutti et al. [49]:

1 $${n}_{{{lens}}}=1.427+(-6.6\times \,{10}^{-5})\times X+(2.35\times \,{10}^{-4})\times \,{X}^{2}$$

where ${n}_{{{lens}}}$ is refractive index and $X$ = age in years–10.

To assess the relative contribution of the anterior and posterior surface shapes at different levels of accommodation demands, the lens shape ratio was calculated as

2 $${{\rm{Lens}}}\,{{\rm{shape}}}\,{{\rm{ratio}}}=\frac{{F}_{{{al}}}}{{F}_{{{pl}}}}$$

where ${F}_{{al}}$ and ${F}_{{{pl}}}$ are the *M* values for the anterior and posterior lens surfaces.

### Statistical Analysis

Statistical analyses were performed using the Statistical Package for the Social Sciences (SPSS version 26.0, ibm.com), Microsoft Excel (Version 2402, microsoft.com) and GraphPad Prism (Version 9.2.0, graphpad.com). Frequency distributions were not significantly different from normality (D’Agostino–Pearson Omnibus test).

To examine the effect of accommodation demand on lens surface parameters *M, J*_0_ and *J*_45_, on *F*_L_ and lens shape ratio between the two refractive groups, as well as any interaction between the two fixed factors (accommodation demand and refractive error group), a linear mixed model (LMM) analysis was used with age as a covariate. To account for the repeated-measures design, subject ID was included as a random intercept. The model was fitted using restricted maximum-likelihood estimation. Significance of main effects was assessed using *F*-tests with the Satterthwaite approximation for degrees of freedom. Model assumptions, including normality and homoscedasticity of residuals and linearity with respect to the relationship with age, were verified by visual inspection of diagnostic plots.

Statistical significance was set at *p* < 0.05. Post-hoc testing was performed using Bonferroni-adjusted pairwise comparisons (see Supplementary file 2).

## Results

### Participants

Table 1 shows participant information and comparisons from the LMM analysis. There was a significant difference in the age of the groups (*F*_1, 304_ = 46.96, *p* < 0.0001), with the myopic group being older by ~1.5 years. To take this into account, all statistical comparisons between groups included age as a covariate. There was no significant variation in age across accommodative demands (*F*_3, 304_ = 0.07, *p* = 0.97), nor was there a significant interaction between refractive error group and accommodative demand on age (*F*_3, 304_ = 0.03, *p* = 0.99), indicating that age differences were consistent across conditions.

**Table 1:** Participant details.

		Accommodation demand (D)				Refractive error group		Accommodation demand		Refractive error group by accommodation demand		
		0	3	6	9	*F*	*p*	*F*	*p*	*F*		*p*
*n*	Non-myopes	73	58	64	57							
	Myopes	18	16	16	10							
Age (years)	Non-myopes	8.6 ± 1.7	8.7 ± 1.7	8.6 ± 1.6	8.6 ± 1.7	46.96	<0.0001*	0.07	0.97	0.03	0.99	
	Myopes	10.1 ± 1.4	10.3 ± 1.4	10.2 ± 1.5	10.4 ± 1.6							
SER (D)	Non-myopes	+0.64 ± 0.35	+0.66 ± 0.36	+0.67 ± 0.36	+0.66 ± 0.37	1223.71	<0.0001*	0.29	0.83	0.29	0.84	
	Myopes	–2.08 ± 0.92	–2.01 ± 0.94	–2.11 ± 0.97	–1.91 ± 1.02							
Accommodation response (D)	Non-myopes	0.0 ± 0.0	+1.49 ± 0.52	+3.92 ± 0.93	+6.62 ± 0.95	0.77	0.38	670.44	<0.0001*	0.40	0.75	
	Myopes	0.0 ± 0.0	+1.65 ± 0.60	+4.14 ± 1.06	+6.57 ± 1.35							

Number of participants (*n*), age and spherical equivalent refraction (SER) in dioptres, presented as mean (±SD) for the refraction groups at each accommodation demand level. *F*-statistics (*F*) and *p*-values (*p*) from the linear mixed model analyses; significant *p*-values indicated by asterisks (*). Accommodation response data taken from Hughes [58].

As expected, SER differed significantly between the refractive error groups. (*F*_1, 304_ = 1223.71, *p* < 0. 0001). There was no main effect of accommodative demand on SER (*F*_3, 304_ = 0.29, *p* = 0.83). There was no significant interaction between refractive error group and accommodation demand on SER (*F*_3, 304_ = 0.28, *p* = 0.84).

As expected, there was a main effect of accommodation demand on accommodative response (*F*_3, 282_ = 670.44, *p* < 0.0001). There was no main effect of refractive group on accommodative response (*F*_1, 282_ = 0.77, *p* = 0.38) nor a significant interaction between refractive group and accommodation demand on accommodative response (*F*_3, 282_ = 0.4, *p* = 0.75).

### Effect of accommodation on lens parameters

**Fig. 2:**
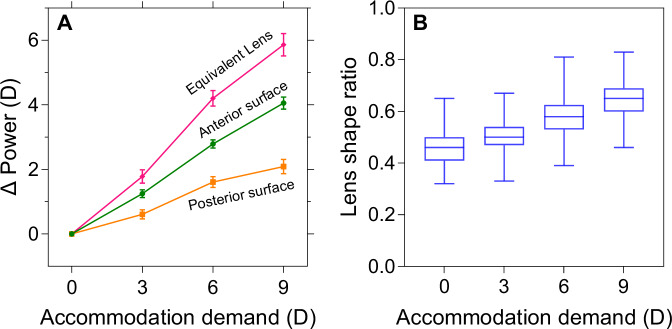
Change in parameters with accommodation demand for all children combined. **A** Anterior surface *M*, posterior surface *M* and *F*_L_; error bars are the standard error of the mean. **B** Lens shape ratio showing median (central horizontal line), interquartile range (upper and lower box limits) and range maxima and minima (error bars), *F*_L_ equivalent lens mean spherical power, *M* spherical equivalent refractive error.

For all children, the baseline mean (±SD) values for anterior and posterior surface *M* and *F*_L_ were +7.73 ± 1.40, +16.68 ± 2.10 and +24.09 ± 2.98 D, respectively (see Supplementary file 2, Table Supp 2.1). With accommodation, the anterior (*F*_3, 304_ = 80.25, *p* < 0.0001) and posterior surface values of *M* (*F*_3, 304_ = 7.26, *p* < 0.0001) and *F*_L_ (*F*_3, 304_ = 34.04, *p* < 0.0001) increased significantly with accommodation demand (Fig. 2A). The mean (±SD) power change for every dioptre of accommodation demand was +0.45 ± 0.17 D for the anterior surface, +0.23 ± 0.20 D for the posterior surface and +0.65 ± 0.32 D for *F*_L_. There was no significant effect of accommodation demand on the astigmatic vectors (*J*_0_ and *J*_45_) for either surface (for anterior, *p* = 0.68 and 0.48; for posterior, *p* = 0.47 and 0.88). All *M* values changed between accommodation demands except for the posterior surface *M* between 6 and 9 D (*t*_57_ = 1.03, *p* = 0.31).

From 0.47 at the 0 D demand, lens shape ratio increased (*F*_3, 304_ = 67.46, *p* < 0.0001) by +0.05 ± 0.05, +0.11 ± 0.06 and +0.17 ± 0.08 for the 3, 6 and 9 D demands, respectively (Fig. 2B).

### Comparison Between Non-myopic and Myopic Children

For the 0 D accommodation demand, non-myopes had higher powers than myopes: anterior surface *M* difference = +1.54 D (*t*_89_ = 4.63, *p* < 0.0001), posterior surface *M* difference = +2.27 D (*t*_89_ = 4.53, *p* < 0.0001) and *F*_L_ difference = +3.69 D (*t*_89_ = 5.38, *p* < 0.0001). Lens astigmatic vectors of the groups were similar except for anterior surface *J*_0_ (*t*_89_ = 2.98, *p* = 0.004). There was no significant difference between the groups for lens shape ratio.

**Fig. 3:**
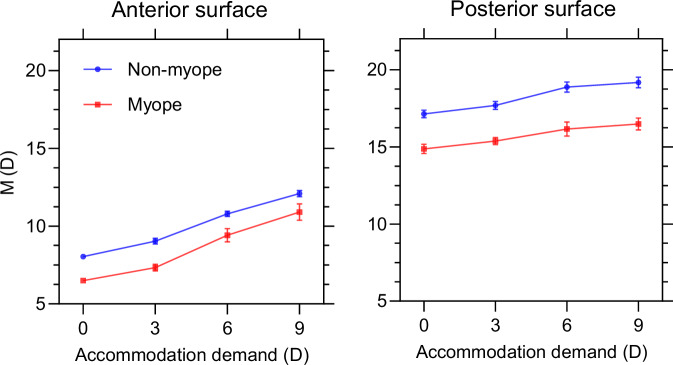
Anterior and posterior surface spherical power, *M*, at different accommodation demands for both refraction groups; error bars are the standard error of the mean.

Across all accommodation demands, the non-myopes had greater anterior surface *M* than the myopes (*F*_1, 304 =_ 48.88, *p* < 0.0001) (Fig. 3). There was no significant interaction between accommodation demand and refractive error group (*F*_3, 304 = _ 0.25, *p* = 0.86), indicating that the rate of change with accommodation was similar for both groups.

Across all accommodation demands, the non-myopes had greater posterior *M* than the myopes (*F*_1, 304 =_ 61.48, *p* < 0.0001 (Fig. 3). There was no significant interaction between accommodation demand and refractive error group (*F*_3, 304 =_ 0.15, *p* = 0.93), indicating that the rate of change with accommodation was similar for both groups.

**Fig. 4:**
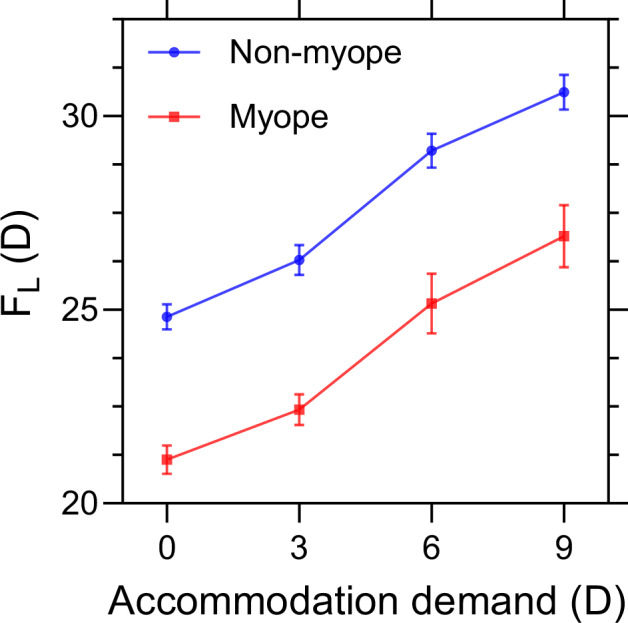
Equivalent lens mean spherical power (*F*_L_) at different accommodation demands for the non-myopic and myopic groups. Error bars are the standard error of the mean.

Across all accommodation demands, the non-myopes had greater *F*_L_ than the myopes (*F*_1, 304 =_ 73.72, *p* < 0.0001) (Fig. 4). There was no significant interaction between accommodation demand and refractive error group (*F*_3, 304 =_ 0.02, *p* = 0.99), indicating the rate of change with accommodation was similar for both groups.

**Fig. 5:**
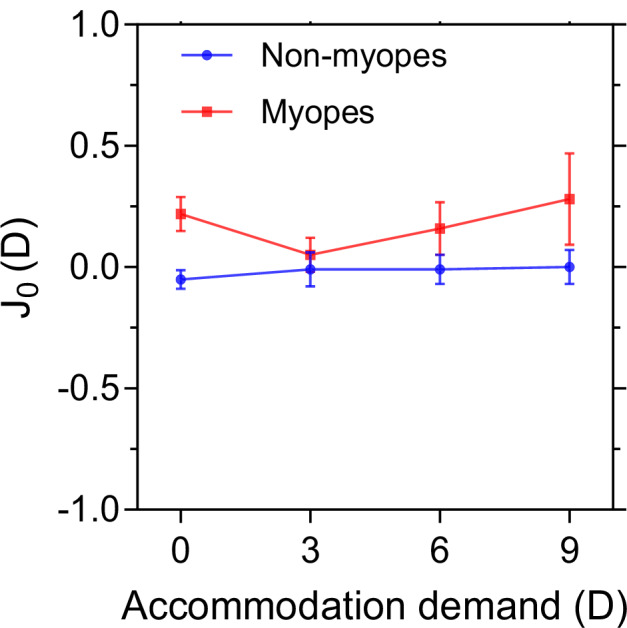
Anterior surface astigmatic vector *J*_0_ at different accommodation demands for the non-myopic and myopic groups. Error bars are the standard error of the mean.

For the astigmatic refractive power vectors, the myopic group exhibited significantly greater anterior lens surface *J*_0_ than the non-myopic group, averaged across all accommodative demands (*F*_1, 304_ = 8.19, *p* = 0.005) (Fig. 5). The difference (estimated marginal mean ± standard error of the mean) was 0.08 ± 0.03 D (95% CI: 0.01–0.15 D). There was no significant interaction between accommodation demand and refractive error group on *J*_0_ (*F*_3, 304 =_ 0.61, *p* = 0.61).

Across all accommodation demands, there was no significant between-group difference in lens shape ratio (*F*_1, 304 =_ 0.83, *p* = 0.36). There was no significant interaction between accommodation demand and refractive error group on lens shape ratio (*F*_3, 304 =_ 1.92, *p* = 0.13); again indicating that the rate of change with accommodation was similar for both groups.

## Discussion

### Accommodation-induced Change in Lens Shape Parameters

To compare with previous studies examining changes in lens parameters during accommodation in adults, the lens surface *M* powers for the non-myopic children were converted to radii of curvature. For the 0 D accommodation demand, the mean (±SD) radii of curvature for the anterior (${r}_{{{\rm{al}}}}$) and posterior (${r}_{{{\rm{pl}}}}$) lens surfaces were 12.2 ± 1.9 and –5.6 ± 0.6 mm, respectively. Previous studies of adults reported ${r}_{{{\rm{al}}}}$ and ${r}_{{pl}}$ ranging from 9.9 to 14.1 mm and from –5.5 to –7.6 mm, respectively, during relaxed accommodation [4, 6–8, 38, 40–42, 44, 45, 47, 50]. The mean ${r}_{{{\rm{al}}}}$ of the children in the current study fell within the mid-range of previously reported adult values, while the mean ${r}_{{{\rm{pl}}}}$ aligns with the lower end of the adult range. This variation in lens measurements could be attributed to two factors: first, children typically have smaller radii of curvature compared to adults [51], and second, the inherent variability in measurements across different instruments used in various studies.

In the current study, mean increases in the anterior and posterior surface *M* per dioptre of accommodation demand were +0.45 and +0.23 D, respectively, or a decrease in the radii of curvature of −0.43 mm/D (range: 0.37–0.49 mm/D) for ${r}_{{{\rm{al}}}}$ and −0.06 mm/D (range: 0.05–0.08 mm/D) for ${r}_{{{\rm{pl}}}}$. This is within the range of values previously reported for adults (${r}_{{{\rm{al}}}}$, 0.3 to 0.93 mm/D, ${r}_{{{\rm{pl}}}}$, 0.04 to 0.2 mm/D) [4, 6–8, 38–43, 45, 50, 52–54]. The mean increase in *F*_L_ of +0.65 D per dioptre of accommodation demand was also similar to studies of young adults using OCT (range: 0.66–0.82 D) [6, 43], but lower than previously reported for young adults using phakometry (1.04–1.09 D) [4, 55]. The increases in anterior surface *M* (+0.40 to +0.69 D) and posterior surface *M* (+0.20 to +0.39 D) per dioptre of accommodation in adults from phakometry measurements [4, 56] were similar to the present study.

As reported previously [38, 52, 56, 57], greater changes were observed for the anterior than the posterior surface *M* with accommodation, so that the lens moved towards an equi-convex shape. No significant difference was observed in the posterior surface power (*M*) between the 6 and 9 D accommodation demands. The posterior lens surface undergoes smaller changes during accommodation, with the anterior surface changes responsible for the dioptric power change at higher accommodation demands [4, 8].

**Fig. 6:**
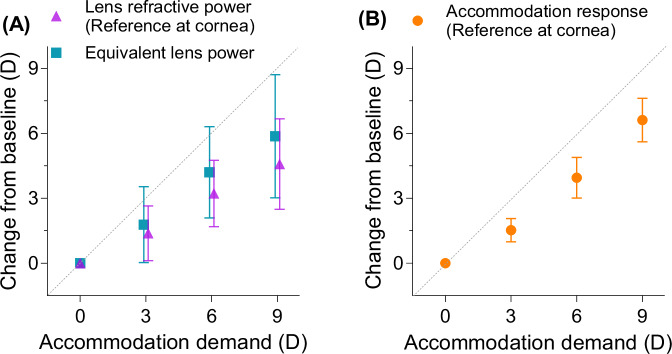
**A** Mean change in equivalent lens power (blue) and lens refractive power referenced at the corneal plane (purple) from baseline (0 D demand) for different accommodation demands, derived from the current study. **B** Mean change in accommodation response from baseline (0 D demand) for the same participants, as previously reported by Hughes [58]. Error bars are the standard deviation.

Participants underaccommodated when viewing through the Badal optometer (Table 1). Accommodative changes relative to the 0 D demand were +1.52, +3.95 and +6.61 D for the 3, 6 and 9 D demands, respectively, as measured by the COAS-HD wavefront sensor (amo-inc.com) (Fig. 6B) [58]. Lens surface curvatures and biometry data were also used to estimate the change in lens refractive power from the 0 D accommodation demand, referenced to the corneal plane by reverse ray tracing. These estimations were compared with the accommodation response measured at the corneal plane. The changes in *F*_L_ and lens refractive power are shown in Fig. 6A. The lens refractive power increased by a mean (±SD) of +1.4 ± 1.3, +3.2 ± 1.5 and +4.6 ± 2.1 D for accommodation demands of 3, 6 and 9 D, respectively. Hughes et al. [10] attributed such accommodation errors to the absence of proximity cues and the limited field of view within the Badal optometer [59].

Lens surface astigmatism *J*_0_ and *J*_45_ remained stable with increasing levels of accommodation, which contrasts with an overall increase in WTR astigmatism of the eye reported previously [14–17]. This indicates that the accommodation-induced WTR shift in ocular astigmatism is unlikely to be driven by changes in lens surface toricity, but rather involves corneal, pupil or other optical alignment effects. Two studies have measured the rate of change in ocular astigmatism with accommodation using aberrometry estimates of refractive error in young adults. Radhakrishnan and Charman [16] found a change of –0.036 DC at axis 176° per dioptre of accommodation demand, while Cheng et al. [17] reported –0.021 DC at axis 180° per dioptre of accommodation demand. Hughes et al. [15] found a change of −0.011 DC at axis 18° per dioptre of accommodation demand in the children reported in the present study. Lens tilt was not evaluated in the current study, but it has been indicated that increased lens tilt may occur during accommodation and could contribute to changes in ocular refraction [16]. Future studies should investigate this factor and its impact on lenticular astigmatism.

### Comparison Between Non-myopic and Myopic Children for Accommodation Induced Change in Lens Shape

Lens surface shapes differed between non-myopic and myopic children. During relaxed accommodation (0 D demand), the non-myopes had higher lens powers with steeper surfaces than myopes, with mean differences (non-myopes–myopes) of +1.54, +2.27 and +3.69 D for anterior surface *M*, posterior surface *M* and *F*_L_, respectively, similar to previously reported findings [24–28]. Lens surface astigmatism was significantly different for the anterior surface *J*_0_ only. Lens shape ratio was also similar between the groups at 0 D accommodation demand.

This is the first study to compare lens powers between non-myopic and myopic children during accommodation. Significant between-group differences were observed in the anterior and posterior surface *M* values, with myopic children displaying flatter anterior and posterior lens surfaces across all accommodation demands compared to the non-myopes. Furthermore, *F*_L_ differed significantly between non-myopic and myopic children, with myopic children displaying lower lens power across all accommodation demands.

The astigmatic lens vectors were similar between non-myopes and myopes, except for the anterior surface *J*_0_, which was marginally greater for myopes across all accommodation demands. Hughes et al. [18] observed a greater astigmatic vector (*J*_0_), i.e., more WTR ocular astigmatism, in myopes across all accommodation stimuli, but compared the 18 myopes included in this study with an age- and sex-matched subgroup of non-myopes. While the anterior lens astigmatic vector *J*_0_ increase was greater in the myopic group during accommodation, the magnitude of these changes (~0.08 D) was small in comparison with the larger changes seen in the lens surface powers (*M*). The magnitude of change in lens surface astigmatism may be too small for the image analysis technique to detect and measure accurately [47], and the limited number of myopic samples at high accommodative demands potentially restricts the conclusions that can be drawn from this finding.

The lens shape ratio increased in both refractive error groups with accommodation, with a similar rate of change, despite the myopic group having a lower lens surface *Ms* than the non-myopic group. Furthermore, the lack of interaction between the refractive error group and accommodation demand for all of the measured lens parameters implies that the effect of accommodation demand on lens parameters was comparable between the groups. This agrees with the finding of similar accommodation responses in both refractive groups across the accommodation demands tested in this study (Table 1).

It is difficult to collect accurate data from children, who may not sustain focus, fixation and precise alignment during the measurement process. This is a potential limitation of the study. In addition, the analysis of lens shape was restricted by decreasing pupil size during the accommodative task [60]. As noted above, participants displayed a lag of accommodation, which may be an unavoidable consequence of taking such measurements in children [61]. Future research utilising simultaneous measurements of accommodation stimulation/response [62] along with ocular biometry would allow a direct comparison between the accommodation stimulus/response and lens shape measurements. This approach could enhance the precision of accommodation-related biometric measurements. Another limitation is the small sample size for the myopic group, particularly at the 6 and 9 D demands. This was mainly due to the greater difficulty in sustaining high accommodative effort among myopic participants, as well as the challenges of obtaining high-quality OCT images during tasks involving large accommodative demands. This disparity in group sizes and attrition within the myopic group at high demands limits the statistical power and precision of the between-group comparisons, particularly for interpreting interaction effects. The missing data in this subgroup may not be completely random, which should be considered when interpreting the results. Consequently, any inference regarding refractive error–dependent differences in accommodation-induced lens shape changes must be made cautiously.

The measurements in this study were based on short-term accommodative tasks, while children are typically exposed to prolonged near tasks at short working distances, ranging from <10 to ~25 cm [21]. Additionally, the calculations for lens powers assumed a uniform refractive index of the lens across all accommodation demands, yet some evidence suggests that there is a small increase in lens equivalent refractive index with accommodation [41, 62, 63], although other studies have reported the changes as negligible [56, 63, 64].

Although differences in lens shape were observed between non-myopic and myopic children in this experiment, no significant differences were found in lens shape changes during accommodation. To validate these primary findings, an additional age- and sex-matched analysis was performed using equal-sized subgroups (*n* = 18 per group). Results were consistent with the main analysis (see Supplementary file 3), supporting the overall conclusions despite the limitations due to a smaller myopic sample. However, the cross-sectional design of this study does not allow for conclusions regarding causality. Given that myopia development and progression are influenced by genetics, environmental factors and ocular growth dynamics [65], the results of this study warrant confirmation through longitudinal studies.

In conclusion, non-myopic children had more powerful lens surfaces and higher lens power than myopic children, and these differences were maintained with increasing accommodation. While the pattern of change with accommodation was similar between refractive groups, the limited myopic sample, particularly at higher accommodation demands, necessitates cautious interpretation of between-group comparisons. Future longitudinal studies with larger and more balanced refractive groups during sustained near tasks are required to confirm these findings and clarify their role in myopia development.

## Supplementary information

Supplementary file

Supplementary file

Supplementary file

Supplementary information

## Data Availability

No datasets were generated or analysed during the current study.
