# Supplementary material for: Crystalline Lens Shape During Accommodation in Children
Source: Ophthalmic Physiol Opt. 2026 Apr 17;46(3):494–501. doi: 10.1007/s44402-026-00069-5 (PMC13369653; doi:10.1007/s44402-026-00069-5)
Supplement: Supplementary file 1 — Supplementary file [file 44402_2026_69_MOESM1_ESM.docx]

# Supplementary file 1

This document details the methodology used for OCT image analysis, outlines the equations used for lens power calculations and repeatability of OCT-based measurements of the lens powers.

## Estimation of lens surface radii of curvature from IOLMaster 700 B-scan images

The IOLMaster 700 produces spatially distorted cross-sectional OCT images. By converting the distorted images back to the optical path distance maps, the real surface shape can be calculated. Six cross-sectional images were analysed to determine radii of curvature for each orientation as follows.

The raw images from the IOLMaster 700 were exported by enabling the raw image export option, located under the "Measurement" settings in the "Advanced Settings" menu of the device. These images were then saved directly to an external hard drive for further analysis.

### **Estimation of radii of curvature for ocular surfaces**

For each measurement of an individual’s axial dimensions, the IOLMaster 700 produces six radial B-scan images, corresponding to meridians of 180°, 30°, 60°, 90°, 120° and 150°. These OCT images are based on optical path maps and contain optical distortions that require correction to estimate the actual shape of ocular surfaces. The images were analysed using custom software in MATLAB (Version 9.9.0.1524771, The Mathworks, Inc., Natick, MA, USA) to determine lens surface radii of curvature values for each orientation of the six B-scan images. The successive steps involved in the image processing are outlined below:

1. **Image segmentation:**

Images were pre-marked using Microsoft Paint (Microsoft Windows 10, Version 21H2, © Microsoft Corporation) to aid image processing algorithms. Small horizontal reference lines marked the anterior lens surface just inside the pupil margin (if visible), to confine analysis to within the pupil, and a mark was made slightly behind the posterior lens (~5 pixels) to assist location of the posterior lens surface (Figure Supp 1.1, upper frame). Limits were marked for the cornea to restrict the analysis, especially for areas affected by eyelid or other artefacts which made sections of the cornea unable to be accurately analysed. Additionally, identifiable artefacts in the images that can be utilised to correct for proprietary distortions were marked with a vertical line. An example of the surfaces marked up is shown in Figure Supp 1.1 (upper frame). Image speckle was reduced by setting pixels to the median value of the 5 pixels it was centred on and those pixels with brightness values less than 20 were discarded. Image segmentation was performed using a pixel threshold increment algorithm to locate the points on each of the 4 surfaces (anterior cornea, posterior cornea, anterior lens, and posterior lens) (Figure Supp 1.1 lower frame).


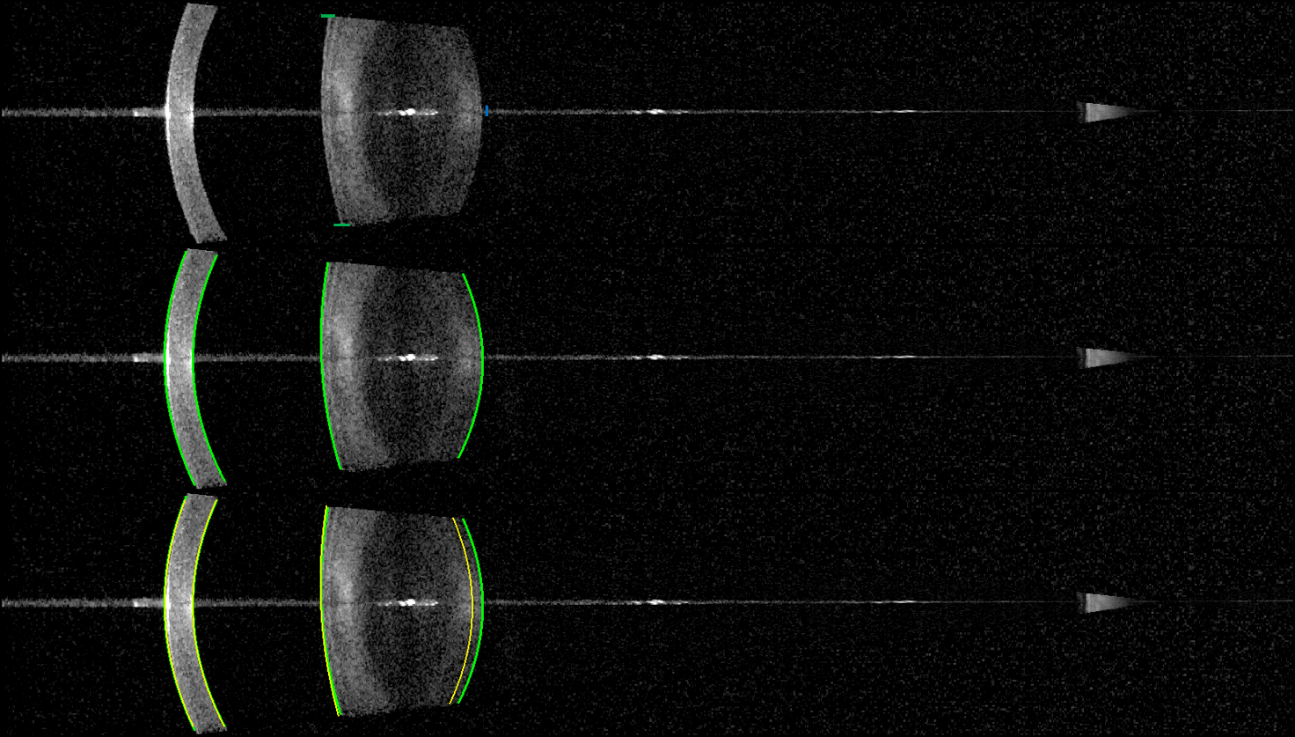


Figure Supp 1.1. An example of a meridional B-scan image from IOLMaster 700. The upper frame shows the marked-up image before segmentation with green lines indicating the anterior lens surface within the pupil margin and a blue line for locating the posterior lens surface. The lower frame illustrates the segmented image with an example of points (green) found for corneal and lenticular surfaces.

1. **Image distortion correction:**

The B-scan image from the IOLMaster 700 is a distorted map of optical path distances in the eye, for different entering ray heights. Artefacts in the image can be used to remove these distortions and provide a map of optical path distances (OPDs) for structures corresponding to different entry ray heights (y’). The map of these OPDs is shown in Figure Supp 1.2.


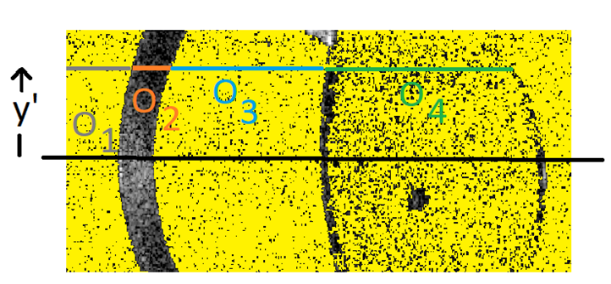


Figure Supp 1.2. Optical path distances for different entering ray heights (*y’*). O_1_, O_2_, O_3_ and O_4_ represent the optical path lengths through air, cornea, aqueous humour, and lens.

For a structure on the posterior lens the optical path distance is calculated using the following equation:

${OPD}_{pl}=O_{1}+O_{2}+O_{3}+O_{4}=n_{air}d_{1}+n_{cornea}d_{2}+n_{aqueous}d_{3}+n_{lens}d_{4}$ (1.1)

where O represents the optical path length in each medium, d is the true distance travelled by the ray through each medium and $n_{air}$, $n_{cornea}$, $n_{aqueous}$, and $n_{lens}$ are the group refractive indices for the air, cornea, aqueous humour, and lens, respectively.

**iii. Determination of actual surface position:**

The positions of the anterior and posterior cornea and lens surfaces were determined using ray tracing. For the IOLMaster 700, which uses a tuneable laser light source (peak λ = 1055 nm), the refractive indices for ray tracing were calculated based on the LeGrand schematic eye indices at 555 nm, adjusted for the 1055 nm light source using the equations from Cooke et al. (2020) and the Atchison & Smith chromatic dispersion model (Atchison & Smith, 2005).

Phase refractive indices $n_{air}$ = 1.0000; $n_{cornea}$ = 1.367574; $n_{aqueous}$ = 1.327942; $n_{lens}$ = 1.408534 were used to calculate the directional changes of the ray at each optical surface, while group refractive indices $n_{air}$ = 1.0000; $n_{cornea}$ = 1.376440; $n_{aqueous}$ =1.336833; $n_{lens}$ = 1.418619 were used to convert the optical path distances to geometrical distances between surfaces, providing estimates of the actual positions of these surfaces.

The position of the posterior corneal surface was calculated by ray tracing through the anterior corneal surface, described by a second order polynomial function fitted with least squares regression to the anterior corneal segmented points, for each entry ray of height $y^{'}$. The refracted ray then propagates through the cornea, traveling a geometrical distance equivalent to the total optical path length, as illustrated in Figure Supp 1.3. The endpoint of this optical path provides an estimate of a point position ($x_{pc}$, $y_{pc}$) on the posterior corneal surface. This relationship is mathematically represented as:

$n_{air}$ $\sin i$ = $n_{cornea}$ $\sin i'$ (1.2)

$d_{1}$ + $d_{2}$ = $O_{1}$/$n_{air}$ + $O_{2}$/$n_{cornea}$ (1.3)


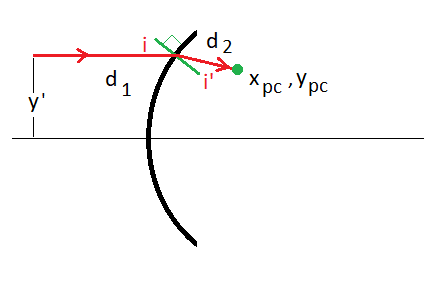


Figure Supp 1.3. Ray tracing through the anterior corneal surface to determine the posterior corneal surface position for $x_{pc}$, $y_{pc}$_._ d_1_ and d_2_ are distances travelled by the entering ray height ($y^{'}$) through air and cornea. *i* and *i’* are angle of incidence and angle of refraction.

**iv. Calculation of anterior and posterior radii of curvature for cornea and lens:**

A second-order polynomial fit was performed for each surface and outlier points were discarded if they exceeded more than two standard deviations from the residuals of these fits (Figure Supp 1.4). The anterior corneal shape was estimated using least squares fit of a second-order polynomial to the distance of the optical path length, represented as:

$x_{ac}=a_{ac}{y'}^{2}+b_{ac}y^{'}+c_{ac}$ (1.4)

The anterior corneal radius of curvature ($r_{ac}$) was determined using the paraxial curvature relationship, as described by Fincham & Freeman (1980, p. 382).

$r_{ac}$ $\cong$ 0.5/$a_{ac}$ (1.5)

Subsequently, once the position of the second order polynomial describing the anterior corneal surface was determined, ray tracing was repeated in a similar fashion to define the posterior corneal surface. The posterior corneal curvature was determined by fitting a second order polynomial to $y_{pc}$ plotted against $x_{pc}$.

$x_{pc}=a_{pc}y_{pc}^{2}+b_{pc}y_{pc}+c_{pc}$ (1.6)

$r_{pc}$ $\cong$ 0.5/$a_{pc}$ (1.7)

Once the posterior corneal surface was defined by a polynomial, similar ray tracing was repeated using the anterior and posterior corneal polynomials to define the surfaces. This process was extended to estimate the points $x_{al}$, $y_{al}$ on the anterior lens surface, with a second-order polynomial fit used to calculate $r_{al}$, the radius of curvature of the anterior lens.

$x_{al}=a_{al}y_{al}^{2}+b_{al}y_{al}+c_{al}$ (1.8)

$r_{al}$ $\cong$ 0.5/$a_{al}$ (1.9)

Using the polynomials for the anterior and posterior cornea and the anterior lens, ray tracing was extended to estimate the points on the posterior lens surface, $x_{pl}$, $y_{pl}$. A second-order polynomial fit was then used to calculate $r_{pl}$, the radius of curvature of the posterior lens.

$x_{pl}=a_{pl}y_{pl}^{2}+b_{apl}y_{pl}+c_{pl}$ (1.10)

$r_{pl}$ $\cong$ 0.5/$a_{pl}$ (1.11)


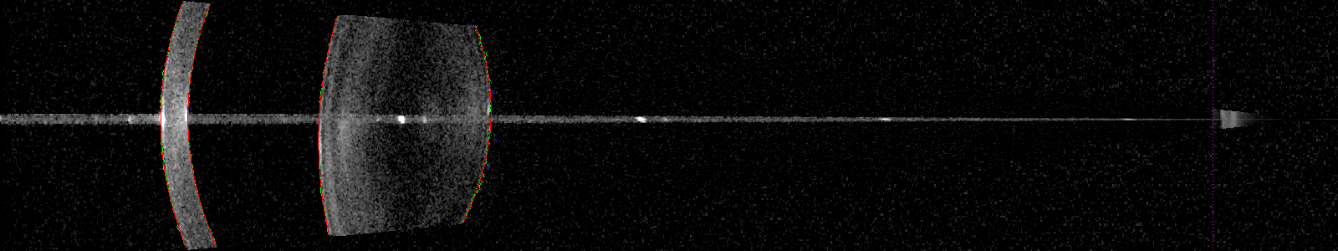


Figure Supp 1.4. An example of a B-scan image after marking the corneal and lenticular surfaces by the software. Fitted points are shown in red, green shows preliminary polynomial fits and blue shows discarded outliers.

### **Estimating toricity for ocular surfaces**

For a toric surface, as illustrated in Figure Supp 1.5, the radius of curvature ($r_{\theta}$) along a meridian at an angle $\theta$ from the meridian with the smallest radius of curvature can be determined from the following relationships:

$C_{\theta}=\frac{\left( C_{max}+C_{min} \right)}{2}+\frac{\left( C_{max}-C_{min} \right)}{2}\mathrm{Cos}\left( 2\theta\right)$ (1.12)

where$C_{\theta}=\frac{1}{r_{\theta}}$,$C_{min}=\frac{1}{r_{min}}$, and $C_{max}=\frac{1}{r_{max}}$ (1.13a, b, c)

where $r_{min}$ and $r_{max}$ are the minimum and maximum radii of curvature, respectively.


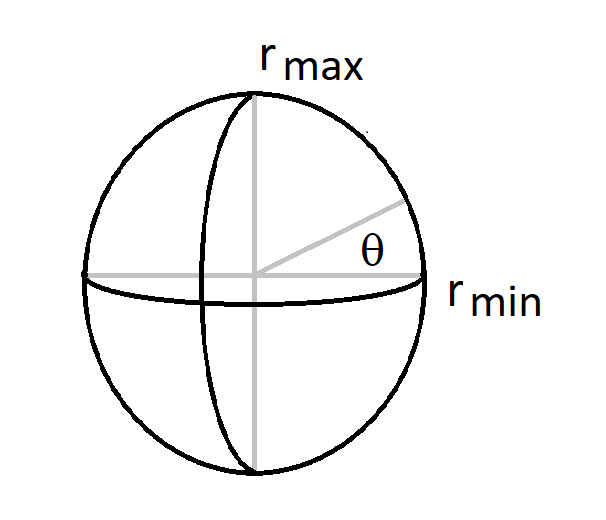


Figure Supp 1.5. Radii of curvature on a toric surface.

For each surface assessed (anterior cornea, posterior cornea, anterior lens, and posterior lens), radii of curvature were obtained for the following meridians $(\phi$): 180°, 30°, 60°, 90°, 120° and 150°. These were converted into curvature ($C=1/r$) and then a cosine least squares fit was applied to the data with the following relationship:

$C=amp\mathrm{Cos} (2(\phi-\phi_{max})+C_{ave}$ (1.14)

where $\phi_{max}$is the meridian with maximum curvature (or minimum radius of curvature), $C_{ave}$ is the average curvature, and “amp” (amplitude) denotes the extent of curvature variation around the mean. The fitting parameters: amp, $\phi_{max}$and $C_{ave}$ were used to estimate the radii of curvature of the principal meridians.

$r_{min}=\frac{1}{(C_{ave}+amp)}$, $r_{max}=\frac{1}{{(C}_{ave}-amp)}$ (1.15a, b)

An example of such a fit is shown in Figure Supp 1.6 for the anterior lens of a participant with $r_{max}$= 15.36 mm; $r_{min}$ = 12.87 mm and $\phi_{max}$= 98°


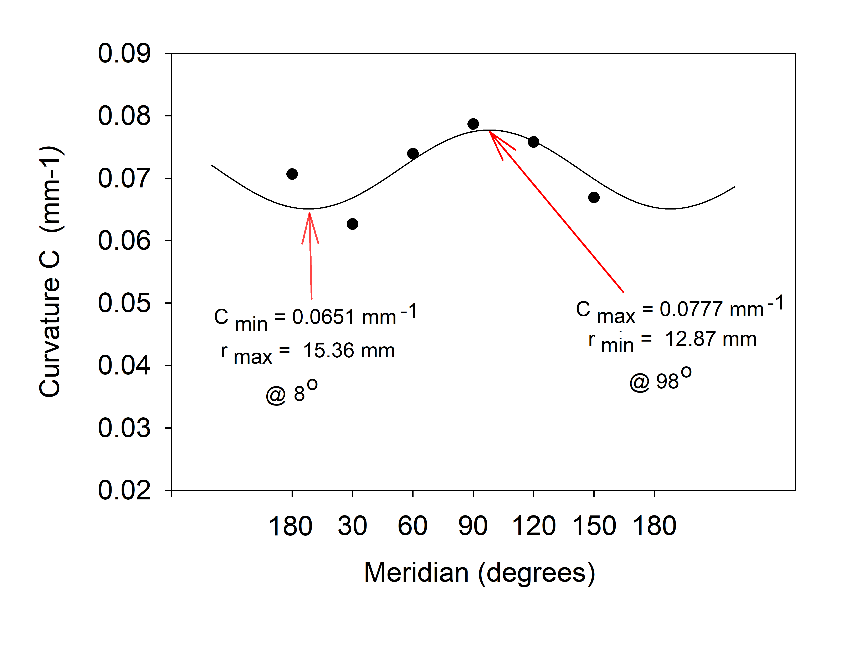


Figure Supp 1.6. An example of a cosine fit for the anterior lens surface measurement from a participant

## Calculation of lens surface powers, equivalent lens power, and lens shape ratio

Using the radii of curvature estimates from the lens shape measures, the mean spherical equivalent refractive power (*M*) vector, and horizontal/vertical (*J_0_*) and oblique astigmatic power (*J_45_*) vectors (Thibos et al., 1997) were determined for the anterior and posterior lens surfaces, using equations 1.16, 1.17, and 1.18. The assumed refractive indices were *n_aqueous_ = n_vitreous_* = 1.336 and *n_L_*=1.422 (Rabbetts, 2007, pp. 450-451). A negative *J_0_* vector for a lens surface would add with-the-rule (WTR) astigmatism to the eye, i.e. it would be corrected by a negative cylinder lens in front of the eye with axis of 180°.

*M* = (*F_max_ + F_min_*) / 2 (1.16)

*J_0_* = $- \frac{C}{2} \times Cos2\alpha$ (1.17)

*J_45_* = $- \frac{C}{2} \times Sin2\alpha$ (1.18)

where *F_max_* and *F_min_* are the maximum and minimum powers for the surface, *C* is the negative cylindrical power and *α* is the steepest meridian.

Equivalent lens mean spherical power (*F_L_*) was calculated using the following equation:

$F_{L}= F_{al}+F_{pl}-\frac{LT}{n_{lens}} \times F_{al}\times F_{pl}$ (1.19)

where $F_{al}$ *and* $F_{pl}$ are anterior and posterior lens surface mean spherical equivalent power (*M*) vectors, $n_{lens}$ is the refractive index of the lens and LT is lens thickness.

Lens shape ratio was calculated as follows:

Lens shape ratio = $\frac{F_{al}}{F_{pl}}$ (1.20)

## Repeatability and reproducibility of IOLMaster 700 measurements

We used the lens power measurements from OCT images taken by two examiners to evaluate the repeatability and reproducibility of the image analysis method (Pradhan, 2025. <https://eprints.qut.edu.au/255818>). The IOLMaster 700 lens power measurements showed high intra-examiner repeatability for both examiners. For the anterior surface power (*M*), the 95% LoA were narrow (approximately ±0.30 D) with strong correlation (R > 0.96) for both examiners. The posterior surface power (*M*) measurements showed slightly wider LoA (~ ±0.80 D). and high correlations (R > 0.91). *FL* also demonstrated high intra-examiner repeatability, with LoA (~ ±0.70 D) and strong correlations (R > 0.96) across examiners.

Inter-examiner measurement comparisons demonstrated excellent agreement between the two examiners. The anterior lens surface power (*M*) measurements showed narrow LoA (±0.22 D) and were strongly correlated (R = 0.99). The LoA for posterior lens surface power (*M*) measurements were slightly wider (±0.64 D) with a high correlation coefficient (R = 0.94). *FL* measurements also showed strong agreement between examiners (LoA ±0.71 D, R = 0.96).
