# Supplementary material for: Crystalline Lens Shape During Accommodation in Children
Source: Ophthalmic Physiol Opt. 2026 Apr 17;46(3):494–501. doi: 10.1007/s44402-026-00069-5 (PMC13369653; doi:10.1007/s44402-026-00069-5)
Supplement: Supplementary file 2 — Supplementary file [file 44402_2026_69_MOESM2_ESM.docx]

## Supplementary file 2

This document includes a table comparing lens parameters between the non-myopic main group (n = 76) and the myopic group (n = 18), with associated statistical outputs corresponding to those presented in the main manuscript. It also provides post-hoc test results between the two refractive groups, as well as an analysis of the relationship between axial length and accommodation-induced changes in lens powers.

Table Supp 2.1. Lens parameters. Mean (± SD) refractive power vectors for the anterior and posterior surfaces, *F_L_*, and lens shape ratio at different accommodation demands for the refraction groups. F-statistics (*F*) and *p*-values (p) from the linear mixed model analyses; significant *p*-values indicated by asterisks (*)

| Refractive power vectors (D) | Refractive error group | 0 D | 3 D | 6 D | 9 D | Refractive error group | | Accommodation demand | | Refractive error group by accommodation demand | |
| --- | --- | --- | --- | --- | --- | --- | --- | --- | --- | --- | --- |
|  |  |  |  |  |  | ***F*** | ***p*** | ***F*** | ***p*** | ***F*** | ***p*** |
| Anterior lens surface | | | | | | | | | | | |
| *M* | Non-myopes | +8.04 ± 1.37 | +9.03 ± 1.48 | +10.78 ± 1.45 | +12.09 ± 1.50 | 48.88 | <0.0001* | 80.25 | <0.0001* | 0.25 | 0.86 |
|  | Myopes | +6.50 ± 0.59 | +7.33 ± 0.86 | +9.41 ± 1.70 | +10.90 ± 1.65 |  |  |  |  |  |  |
|  | All | +7.73 ± 1.40 | +8.66 ± 1.54 | +10.51 ± 1.60 | +11.91 ± 1.57 |  | | | | | |
| *J_0_* | Non-myopes | –0.05 ± 0.36 | –0.01 ± 0.52 | –0.01 ± 0.50 | +0.00 ± 0.54 | 8.19 | 0.005* | 0.51 | 0.68 | 0.61 | 0.61 |
|  | Myopes | +0.22 ± 0.29 | +0.05 ± 0.27 | +0.16 ± 0.46 | +0.28 ± 0.59 |  |  |  |  |  |  |
|  | All | +0.00 ± 0.36 | +0.00 ± 0.47 | +0.02 ± 0.49 | +0.05 ± 0.55 |  | | | | | |
| *J_45_* | Non-myopes | +0.08 ± 0.36 | –0.08 ± 0.47 | +0.04 ± 0.46 | +0.10 ± 0.56 | 0.19 | 0.66 | 0.83 | 0.48 | 0.80 | 0.50 |
|  | Myopes | +0.03 ± 0.32 | +0.03 ± 0.35 | –0.13 ± 0.63 | +0.09 ± 0.47 |  |  |  |  |  |  |
|  | All | +0.07 ± 0.36 | –0.05 ± 0.45 | +0.01 ± 0.50 | +0.10 ± 0.55 |  | | | | | |
| Posterior lens surface | | | | | | | | | | | |
| *M* | Non-myopes | +17.13 ± 2.03 | +17.68 ± 1.96 | +18.87 ± 2.63 | +19.17 ± 2.61 | 61.48 | <0.0001* | 7.26 | <0.0001* | 0.15 | 0.93 |
|  | Myopes | +14.86 ± 1.26 | +15.37 ± 0.92 | +16.15 ± 1.84 | +16.48 ± 1.21 |  |  |  |  |  |  |
|  | All | +16.68 ± 2.10 | +17.18 ± 2.02 | +18.33 ± 2.71 | +18.77 ± 2.63 |  | | | | | |
| *J_0_* | Non-myopes | +0.02 ± 1.17 | +0.22 ± 0.91 | +0.12 ± 1.18 | –0.09 ± 1.46 | 1.09 | 0.30 | 0.84 | 0.47 | 1.02 | 0.39 |
|  | Myopes | +0.26 ± 0.75 | +0.44 ± 0.64 | –0.18 ± 1.00 | +0.46 ± 1.53 |  |  |  |  |  |  |
|  | All | +0.06 ± 1.10 | +0.27 ± 0.86 | +0.06 ± 1.15 | –0.01 ± 1.47 |  | | | | | |
| *J_45_* | Non-myopes | –0.03 ± 1.14 | –0.10 ± 1.30 | +0.17 ± 1.84 | +0.10 ± 1.48 | 0.14 | 0.71 | 0.22 | 0.88 | 0.9 | 0.97 |
|  | Myopes | –0.08 ± 0.94 | –0.13 ± 0.94 | –0.08 ± 0.81 | +0.12 ± 1.58 |  |  |  |  |  |  |
|  | All | –0.04 ± 1.10 | –0.10 ± 1.23 | +0.12 ± 1.68 | +0.10 ± 1.49 |  | | | | | |
| Equivalent lens power (D) | | | | | | | | | | | |
| *F_L_* | Non-myopes | +24.82 ± 2.80 | +26.29 ± 2.95 | +29.11 ± 3.52 | +30.62 ± 3.43 | 13546.39 | <0.0001* | 34.04 | <0.0001* | 0.02 | 0.99 |
|  | Myopes | +21.13± 1.54 | +22.42± 1.60 | +25.16 ± 3.07 | +26.90 ± 2.53 |  |  |  |  |  |  |
|  | All | +24.09 ± 2.98 | +25.46 ± 3.15 | +28.32 ± 3.76 | +30.06 ± 3.56 |  | | | | | |
| Lens shape ratio | Non-myopes | 0.47 ± 0.07 | 0.51 ± 0.06 | 0.58 ± 0.07 | 0.64 ± 0.08 | 0.83 | 0.36 | 67.46 | <0.0001* | 1.92 | 0.13 |
|  | Myopes | 0.44± 0.04 | 0.48± 0.04 | 0.58 ± 0.08 | 0.66± 0.08 |  |  |  |  |  |  |
|  | All | +0.47 ± 0.07 | +0.50 ± 0.06 | +0.58 ± 0.07 | +0.64 ± 0.08 |  | | | | | |

## Post-hoc test results for measured lens parameters

Post-hoc pairwise comparisons for the measured lens parameters (anterior lens powers *M*, *J_0_*, *J_45_*; posterior lens powers *M*, *J_0_*, *J_45_*; equivalent lens power and lens shape ratio) were adjusted using Bonferroni correction to control for multiple comparisons for the non-myopic (n=76) and the myopic (n=18) groups are presented below:

Table Supp 2.2: Anterior lens surface *M*

| Acc Dem (A) | Acc Dem (B) | Mean difference (A-B) | Std. Error | Df | Sig. | 95% Confidence Interval for Difference | |
| --- | --- | --- | --- | --- | --- | --- | --- |
|  |  |  |  |  |  | Lower Bound | Upper Bound |
| 0 D | 3 D | -0.91 | 0.27 | 304 | 0.006 | -1.63 | -0.18 |
|  | 6 D | -2.82 | 0.27 | 304 | 0.000 | -3.54 | -2.10 |
|  | 9 D | -4.22 | 0.31 | 304 | 0.000 | -5.03 | -3.41 |
| 3 D | 0 D | 0.91 | 0.27 | 304 | 0.006 | 0.18 | 1.63 |
|  | 6 D | -1.92 | 0.28 | 304 | 0.000 | -2.66 | -1.17 |
|  | 9 D | -3.32 | 0.31 | 304 | 0.000 | -4.15 | -2.48 |
| 6 D | 0 D | 2.82 | 0.27 | 304 | 0.000 | 2.10 | 3.54 |
|  | 3 D | 1.92 | 0.28 | 304 | 0.000 | 1.17 | 2.66 |
|  | 9 D | -1.40 | 0.31 | 304 | 0.000 | -2.23 | -0.57 |
| 9 D | 0 D | 4.22 | 0.30 | 304 | 0.000 | 3.41 | 5.03 |
|  | 3 D | 3.32 | 0.31 | 304 | 0.000 | 2.48 | 4.15 |
|  | 6 D | 1.40 | 0.31 | 304 | 0.000 | 0.57 | 2.23 |

Table Supp 2.3: Anterior lens surface *J_0_*

| Acc Dem (A) | Acc Dem (B) | Mean difference (A-B) | Std. Error | Df | Sig. | 95% Confidence Interval for Difference | |
| --- | --- | --- | --- | --- | --- | --- | --- |
|  |  |  |  |  |  | Lower Bound | Upper Bound |
| 0 D | 3 D | 0.07 | 0.09 | 304 | 1.00 | -0.17 | 0.31 |
|  | 6 D | 0.01 | 0.09 | 304 | 1.00 | 0.23 | 0.25 |
|  | 9 D | -0.06 | 0.10 | 304 | 1.00 | -0.32 | 0.21 |
| 3 D | 0 D | -0.07 | 0.09 | 304 | 1.00 | -0.31 | 0.17 |
|  | 6 D | -0.06 | 0.09 | 304 | 1.00 | -0.30 | 0.19 |
|  | 9 D | -0.13 | 0.10 | 304 | 1.00 | -0.4 | 0.15 |
| 6 D | 0 D | -0.01 | 0.09 | 304 | 1.00 | -0.25 | 0.23 |
|  | 3 D | 0.06 | 0.09 | 304 | 1.00 | -0.19 | 0.30 |
|  | 9 D | -0.07 | 0.10 | 304 | 1.00 | -0.34 | 0.20 |
| 9 D | 0 D | 0.06 | 0.10 | 304 | 1.00 | -0.21 | 0.32 |
|  | 3 D | 0.13 | 0.10 | 304 | 1.00 | -0.15 | 0.40 |
|  | 6 D | 0.07 | 0.10 | 304 | 1.00 | -0.20 | 0.34 |

Table Supp 2.4: Anterior lens surface *J_45_*

| Acc Dem (A) | Acc Dem (B) | Mean difference (A-B) | Std. Error | Df | Sig. | 95% Confidence Interval for Difference | |
| --- | --- | --- | --- | --- | --- | --- | --- |
|  |  |  |  |  |  | Lower Bound | Upper Bound |
| 0 D | 3 D | 0.07 | 0.09 | 304 | 1.00 | -0.16 | 0.31 |
|  | 6 D | 0.09 | 0.09 | 304 | 1.00 | -0.14 | 0.33 |
|  | 9 D | -0.04 | 0.10 | 304 | 1.00 | -0.31 | 0.22 |
| 3 D | 0 D | -0.07 | 0.09 | 304 | 1.00 | -0.31 | 0.16 |
|  | 6 D | -0.19 | 0.09 | 304 | 1.00 | -0.22 | 0.26 |
|  | 9 D | -0.12 | 0.10 | 304 | 1.00 | -0.39 | 0.15 |
| 6 D | 0 D | -0.09 | 0.09 | 304 | 1.00 | -0.33 | 0.14 |
|  | 3 D | -0.02 | 0.09 | 304 | 1.00 | -0.26 | 0.22 |
|  | 9 D | -0.14 | 0.10 | 304 | 1.00 | -0.41 | 0.13 |
| 9 D | 0 D | 0.04 | 0.10 | 304 | 1.00 | -0.22 | 0.31 |
|  | 3 D | 0.12 | 0.10 | 304 | 1.00 | -0.15 | 0.39 |
|  | 6 D | 0.14 | 0.10 | 304 | 1.00 | -0.13 | 0.41 |

Table Supp 2.5: Posterior lens surface *M*

| Acc Dem (A) | Acc Dem (B) | Mean difference (A-B) | Std. Error | Df | Sig. | 95% Confidence Interval for Difference | |
| --- | --- | --- | --- | --- | --- | --- | --- |
|  |  |  |  |  |  | Lower Bound | Upper Bound |
| 0 D | 3 D | -0.53 | 0.42 | 304 | 1.00 | -1.64 | 0.58 |
|  | 6 D | -1.52 | 0.42 | 304 | 0.002 | -2.63 | -0.41 |
|  | 9 D | -1.83 | 0.47 | 304 | 0.001 | -3.08 | -0.56 |
| 3 D | 0 D | 0.53 | 0.42 | 304 | 1.00 | -0.58 | 1.64 |
|  | 6 D | -0.99 | 0.43 | 304 | 0.14 | -2.23 | 0.16 |
|  | 9 D | -1.30 | 0.48 | 304 | 0.04 | -2.58 | -0.02 |
| 6 D | 0 D | 1.52 | 0.42 | 304 | 0.002 | 0.41 | 2.63 |
|  | 3 D | 0.99 | 0.43 | 304 | 0.14 | -0.16 | 2.13 |
|  | 9 D | -0.31 | 0.48 | 304 | 1.00 | -1.59 | 0.96 |
| 9 D | 0 D | 1.83 | 0.47 | 304 | 0.001 | 0.59 | 3.08 |
|  | 3 D | 1.30 | 0.48 | 304 | 0.044 | 0.02 | 2.58 |
|  | 6 D | 0.31 | 0.48 | 304 | 1.00 | -0.96 | 1.59 |

Table Supp 2.6: Posterior lens surface *J_0_*

| Acc Dem (A) | Acc Dem (B) | Mean difference (A-B) | Std. Error | Df | Sig. | 95% Confidence Interval for Difference | |
| --- | --- | --- | --- | --- | --- | --- | --- |
|  |  |  |  |  |  | Lower Bound | Upper Bound |
| 0 D | 3 D | -0.19 | 0.22 | 304 | 1.00 | -0.78 | 0.40 |
|  | 6 D | 0.17 | 0.22 | 304 | 1.00 | -0.42 | 0.76 |
|  | 9 D | -0.05 | 0.25 | 304 | 1.00 | -0.71 | 0.61 |
| 3 D | 0 D | 0.19 | 0.22 | 304 | 1.00 | -0.40 | 0.78 |
|  | 6 D | 0.36 | 0.23 | 304 | 0.70 | -0.25 | 0.97 |
|  | 9 D | 0.14 | 0.26 | 304 | 1.00 | -0.54 | 0.82 |
| 6 D | 0 D | -0.17 | 0.22 | 304 | 1.00 | -0.76 | 0.42 |
|  | 3 D | -0.36 | 0.23 | 304 | 0.70 | -0.97 | 0.25 |
|  | 9 D | -0.22 | 0.25 | 304 | 1.00 | -0.90 | 0.46 |
| 9 D | 0 D | 0.05 | 0.25 | 304 | 1.00 | -0.61 | 0.71 |
|  | 3 D | -0.14 | 0.26 | 304 | 1.00 | -0.82 | 0.54 |
|  | 6 D | 0.22 | 0.25 | 304 | 1.00 | -0.46 | 0.90 |

Table Supp 2.7: Posterior lens surface *J_45_*

| Acc Dem (A) | Acc Dem (B) | Mean difference (A-B) | Std. Error | Df | Sig. | 95% Confidence Interval for Difference | |
| --- | --- | --- | --- | --- | --- | --- | --- |
|  |  |  |  |  |  | Lower Bound | Upper Bound |
| 0 D | 3 D | 0.06 | 0.27 | 304 | 1.00 | -0.65 | 0.77 |
|  | 6 D | -0.10 | 0.27 | 304 | 1.00 | -0.80 | 0.61 |
|  | 9 D | -0.16 | 0.30 | 304 | 1.00 | -0.96 | 0.63 |
| 3 D | 0 D | -0.06 | 0.27 | 304 | 1.00 | -0.77 | 0.65 |
|  | 6 D | -0.15 | 0.28 | 304 | 1.00 | -0.89 | 0.58 |
|  | 9 D | -0.22 | 0.31 | 304 | 1.00 | -1.04 | 0.60 |
| 6 D | 0 D | 0.10 | 0.27 | 304 | 1.00 | -0.61 | 0.80 |
|  | 3 D | 0.15 | 0.28 | 304 | 1.00 | -0.58 | 0.89 |
|  | 9 D | -0.07 | 0.31 | 304 | 1.00 | -0.88 | 0.75 |
| 9 D | 0 D | 0.16 | 0.30 | 304 | 1.00 | -0.63 | 0.96 |
|  | 3 D | 0.22 | 0.31 | 304 | 1.00 | -0.60 | 1.04 |
|  | 6 D | 0.07 | 0.31 | 304 | 1.00 | -0.75 | 0.88 |

Table Supp 2.8: Equivalent lens power

| Acc Dem (A) | Acc Dem (B) | Mean difference (A-B) | Std. Error | Df | Sig. | 95% Confidence Interval for Difference | |
| --- | --- | --- | --- | --- | --- | --- | --- |
|  |  |  |  |  |  | Lower Bound | Upper Bound |
| 0 D | 3 D | -1.38 | 0.58 | 304 | 0.11 | -2.93 | 0.17 |
|  | 6 D | -0.16 | 0.58 | 304 | 0.000 | -5.70 | -2.62 |
|  | 9 D | -5.79 | 0.65 | 304 | 0.000 | -7.52 | -4.05 |
| 3 D | 0 D | 1.38 | 0.58 | 304 | 0.11 | -0.17 | 2.93 |
|  | 6 D | -2.78 | 0.60 | 304 | 0.000 | -4.37 | -1.18 |
|  | 9 D | -4.40 | 0.67 | 304 | 0.000 | 6.19 | -2.62 |
| 6 D | 0 D | 4.16 | 0.58 | 304 | 0.000 | 2.62 | 5.70 |
|  | 3 D | 2.78 | 0.60 | 304 | 0.000 | 1.18 | 4.37 |
|  | 9 D | -1.63 | 0.67 | 304 | 0.09 | -3.40 | 0.15 |
| 9 D | 0 D | 5.79 | 0.65 | 304 | 0.000 | 4.05 | 7.52 |
|  | 3 D | 4.40 | 0.67 | 304 | 0.000 | 2.62 | 6.19 |
|  | 6 D | 1.63 | 0.69 | 304 | 0.09 | -0.15 | 3.40 |

Table Supp 2.9: Lens shape ratio

| Acc Dem (A) | Acc Dem (B) | Mean difference (A-B) | Std. Error | Df | Sig. | 95% Confidence Interval for Difference | |
| --- | --- | --- | --- | --- | --- | --- | --- |
|  |  |  |  |  |  | Lower Bound | Upper Bound |
| 0 D | 3 D | -0.04 | 0.01 | 304 | 0.03 | -0.07 | -0.003 |
|  | 6 D | -0.12 | 0.01 | 304 | 0.000 | -0.16 | -0.09 |
|  | 9 D | -0.19 | 0.01 | 304 | 0.000 | -0.23 | -0.15 |
| 3 D | 0 D | 0.04 | 0.01 | 304 | 0.03 | 0.003 | 0.07 |
|  | 6 D | -0.08 | 0.01 | 304 | 0.000 | -0.12 | -0.05 |
|  | 9 D | 0.15 | 0.01 | 304 | 0.000 | -0.19 | -0.11 |
| 6 D | 0 D | 0.12 | 0.01 | 304 | 0.000 | 0.09 | 0.16 |
|  | 3 D | 0.08 | 0.01 | 304 | 0.000 | 0.05 | 0.12 |
|  | 9 D | -0.07 | 0.01 | 304 | 0.000 | -0.11 | -0.03 |
| 9 D | 0 D | 0.19 | 0.01 | 304 | 0.000 | 0.15 | 0.23 |
|  | 3 D | 0.15 | 0.01 | 304 | 0.000 | 0.11 | 0.19 |
|  | 6 D | 0.07 | 0.01 | 304 | 0.000 | 0.03 | 0.11 |

## Relationship between axial length and change in lens powers with accommodation in non-myopic and myopic eyes

Figure Supp 2.1. shows the relationship between axial length and change in lens surface powers and equivalent lens power across the three accommodative demands (3 D, 6 D, 9 D), stratified by refractive error group.

For the 3 D demand, correlation analysis revealed weak and non-significant relationships between axial length and changes in anterior lens power (r = –0.081, p = 0.456), posterior lens power (r = 0.087, p = 0.423), and equivalent lens power (r = 0.012, p = 0.912).

For the 6 D demand, no significant correlations were found between axial length and anterior lens power (r = –0.009, p = 0.936), posterior lens power (r = –0.091, p = 0.416), or equivalent lens power (r = –0.057, p = 0.611).

For the 9 D demand, similarly weak and non-significant correlations were observed for anterior lens power (r = 0.128, p = 0.295), posterior lens power (r = –0.016, p = 0.896), and equivalent lens power (r = 0.068, p = 0.579).

Overall, these findings indicate no statistically significant relationship between axial length and changes in lens surface or equivalent powers across the measured accommodation demands.


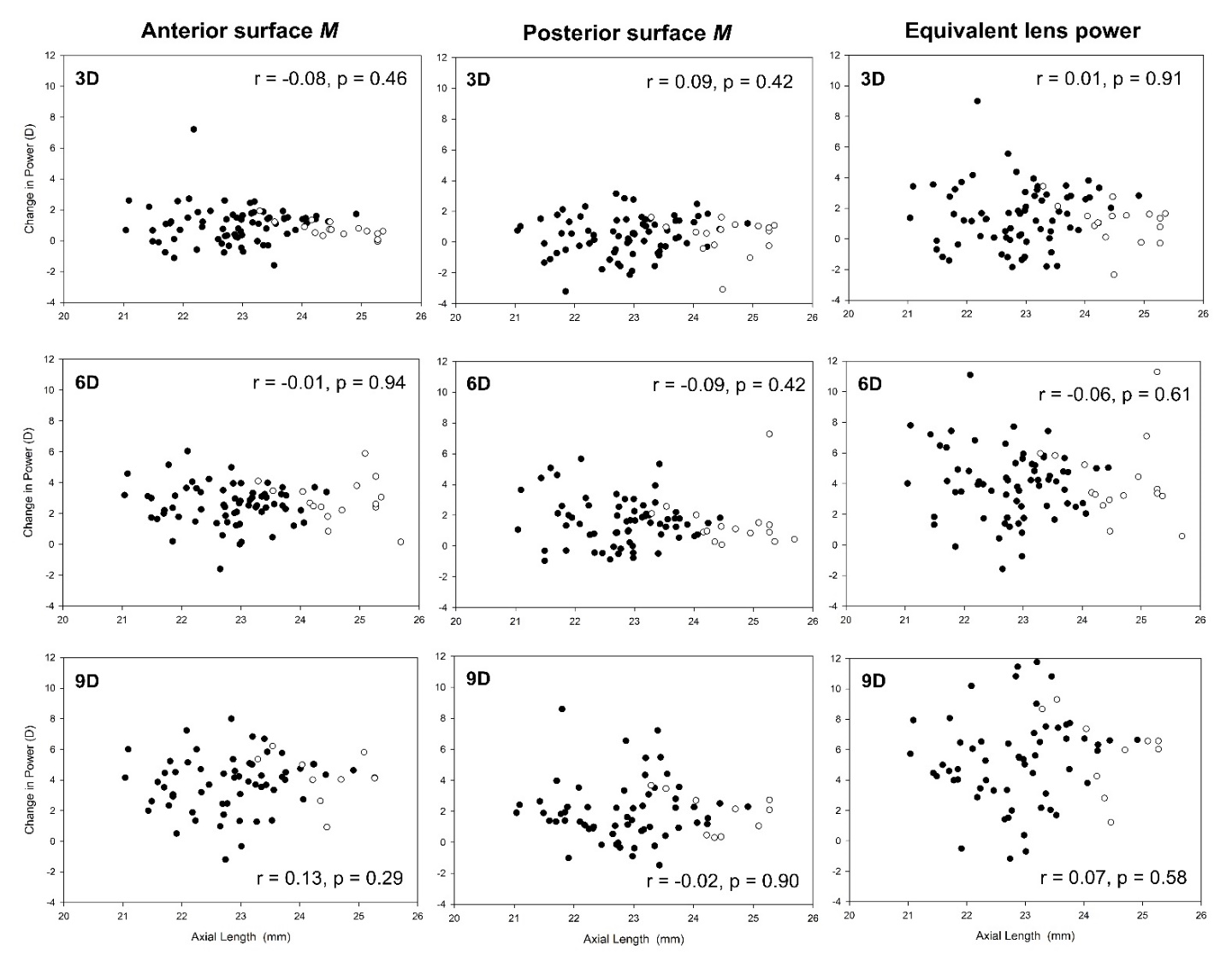


Figure Supp 2.1. Relationship, between axial length (mm) and change in anterior and posterior lens surface power (D) and equivalent lens power (D) at 3 D, 6 D and 9 D accommodation demands. (Solid black circles – non-myopes, open circles – myopes)
