# Supplementary material for: Crystalline Lens Shape During Accommodation in Children
Source: Ophthalmic Physiol Opt. 2026 Apr 17;46(3):494–501. doi: 10.1007/s44402-026-00069-5 (PMC13369653; doi:10.1007/s44402-026-00069-5)
Supplement: Supplementary file 3 — Supplementary file [file 44402_2026_69_MOESM3_ESM.docx]

# Supplementary file 3

This document contains the comparison between the group of myopic children, with a subgroup of age- and sex- matched non-myopic children. This is an alternative analysis to that in the main paper, but in practical terms the results are similar.

## Comparison between age and sex matched non-myopic and myopic children

A group of 18 myopic participants (mean SER –2.1 ± 0.9 D, range –3.50 to –0.75 D), with a mean (± SD) age of 10.1 ± 1.4 years (range 7.3–12.7 years) was compared with a subgroup of 18 age and sex-matched non-myopic participants [mean SER +0.6 ± 0.2 D, range +0.25 to +0.88 D] [mean age 10.1 ± 1.1 years, range 7.3–11.6 years]. All participants had astigmatism of ≤ 0.75 DC magnitude. The mean ages for the groups were similar [independent samples t-test, t_(34)_ = 0.02, p = 0.98]. Table Supp 3.1 shows the sample sizes, mean (± SD) ages and SER of the non-myopic and myopic refractive error groups for each accommodation demand (0, 3, 6 and 9 D). There were no significant differences in age [F_(3, 119)_ = 0.03, p = 0.99] or SER [F_(3, 119)_ = 0.13, p = 0.94] within each refractive error group at any accommodation demand, nor a significant interaction between refractive error group and accommodation demand for SER [F_(3, 119)_ = 0.1, p = 0.96] or for age [F_(3, 119)_ = 0.06, p = 0.98]. The accommodative response did not differ between the refractive groups across all accommodation demands [F_(1, 35.3)_ = 0.07, p = 0.79], nor was there a significant interaction between refractive groups and accommodation demand for accommodative response [F_(3, 81.82)_ = 0.81, p = 0.49]. The sample size varied with accommodation demand and refractive group due to poor image quality of some IOL Master images as outlined in the manuscript.

Table Supp 3.1. Sample size, age and spherical equivalent refraction (SER) presented as mean (± SD) for non-myopic and myopic participants at each accommodation demand. F-statistics and p-values (p) from the linear mixed model analyses; significant *p*-values indicated by asterisks (*). Accommodation response data were taken from Hughes et al. (2022).

|  |  | Accommodation demand (D) | | | | *F* | *p* |
| --- | --- | --- | --- | --- | --- | --- | --- |
|  |  | **0** | **3** | **6** | **9** |  |  |
| Sample size | Non-myopes | 18 | 18 | 16 | 15 |  |  |
|  | Myopes | 18 | 16 | 16 | 10 |  |  |
| Age (years) | Non-myopes | 10.1 ± 1.1 | 10.1 ± 1.1 | 10.1 ± 1.2 | 10.1 ± 1.2 | 0.21 | 0.65 |
|  | Myopes | 10.1 ± 1.4 | 10.3 ± 1.4 | 10.2 ± 1.5 | 10.4 ± 1.6 |  |  |
| SER (D) | Non-myopes | +0.6 ± 0.2 | +0.6 ± 0.2 | +0.6 ± 0.2 | +0.6 ± 0.2 | 481.71 | <0.0001* |
|  | Myopes | –2.1 ± 0.9 | –2.0 ± 0.9 | –2.1 ± 1.0 | –1.9 ± 1.0 |  |  |
| Accommodation response (D) | Non-myopes | 0.0 ± 0.0 | +1.5 ± 0.5 | +4.2 ± 0.8 | +6.9 ± 1.1 | 695.79 | <0.0001* |
|  | Myopes | 0.0 ± 0.0 | +1.7 ± 0.6 | +4.1 ± 1.0 | +6.6 ± 1.4 |  |  |

D = Diopter

Table Supp 3.2 shows the power vectors of the lens surfaces, the mean equivalent lens powers (*F_L_*), and the lens shape ratios for both refractive error groups at the different accommodation demands.

At the 0 D accommodation demand, the non-astigmatic mean powers were higher for the non-myopes than for the myopes: anterior surface *M* [t_(34)_ = 2.3, p = 0.028]; posterior surface *M* [t_(34)_ = 2.88, p = 0.007]; *F_L_* [t_(34)_ = 3.12, p = 0.004]. Lens shape ratios were not significantly different between the two groups [t_(34)_ = 0.4, p = 0.69]. For the astigmatic power vectors, only the anterior surface *J_0_* was significantly different between the two groups [t_(34)_ = 3.04, p = 0.005] with a mean difference of 0.36 D indicating a greater WTR astigmatism from the anterior lens surface in myopes compared to non-myopes

The anterior surface *M* increased significantly with increasing accommodation demand [F_(3, 119)_ = 8.3, p < 0.001] with a significant between-group difference [F_(1, 119)_ = 14.75, p < 0.001] and, with the non-myopes exhibiting greater powers than the myopes across all accommodation demands (Figure Supp 3.1). The change in anterior surface *M* with accommodation was similar for the refractive error groups [F_(3, 119)_ = 0.18, p = 0.91].

The posterior surface *M* increased significantly [F_(3, 119)_ = 3.88, p = 0.011] with increasing accommodation demand, and there was a significant between-group difference, with the non-myopes having greater powers across all accommodation demands [F_(1, 119)_ = 36.36, p < 0.001] (Figure Supp 3.1). There was no significant interaction between accommodation demand and refractive error group [F_(3, 119)_ = 0.76, p = 0.52], indicating that the change in posterior surface *M* with accommodation was similar for myopic and non-myopic children.

Table Supp 3.2. Mean (± SD) refractive power vectors for the anterior and posterior surfaces (*M, J_0_, and J_45_*), mean equivalent lens power (*F_L_*), and mean lens shape ratio for the two refractive error groups and at the different accommodation demands for the myopic and non-myopic groups. F-statistics and p-values (p) from the linear mixed model analyses; significant *p*-values indicated by asterisks (*).

| Refractive power vectors (D) | Refractive error group | 0 D | 3 D | 6 D | 9 D | Refractive error group | | Accommodation demand | | Refractive error group by accommodation demand | |
| --- | --- | --- | --- | --- | --- | --- | --- | --- | --- | --- | --- |
|  |  |  |  |  |  | ***F*** | ***p*** | ***F*** | ***p*** | ***F*** | ***p*** |
| Anterior lens surface | | | | | | | | | | | |
| *M* | Non-myopes | +7.17 ± 1.20 | +8.44 ± 1.21 | +10.30 ± 1.10 | +11.72 ± 1.62 | 14.75 | <0.001* | 8.30 | <0.001* | 0.18 | 0.91 |
|  | Myopes | +6.50 ± 0.59 | +7.33 ± 0.86 | +9.41 ± 1.70 | +10.90 ± 1.65 |  |  |  |  |  |  |
| *J_0_* | Non-myopes | –0.14 ± 0.41 | –0.05 ± 0.53 | –0.01 ± 0.57 | +0.04 ± 0.54 | 6.90 | 0.01* | 0.43 | 0.73 | 0.51 | 0.67 |
|  | Myopes | +0.22 ± 0.29 | +0.05 ± 0.27 | +0.16 ± 0.46 | +0.28 ± 0.59 |  |  |  |  |  |  |
| *J_45_* | Non-myopes | +0.17 ± 0.41 | –0.09 ± 0.26 | +0.08 ± 0.41 | +0.11 ± 0.40 | 0.00 | 1.00 | 1.23 | 0.30 | 0.00 | 1.00 |
|  | Myopes | +0.03 ± 0.32 | +0.03 ± 0.35 | –0.13 ± 0.63 | +0.09 ± 0.47 |  |  |  |  |  |  |
| Posterior lens surface | | | | | |  |  |  |  |  |  |
| *M* | Non-myopes | +16.03 ± 1.19 | +16.72 ± 0.93 | +17.42 ± 1.08 | +18.68 ± 2.17 | 36.36 | <0.001* | 3.88 | 0.01* | 0.76 | 0.52 |
|  | Myopes | +14.86 ± 1.26 | +15.37 ± 0.92 | +16.15 ± 1.84 | +16.48 ± 1.21 |  |  |  |  |  |  |
| *J_0_* | Non-myopes | +0.01 ± 0.66 | 0.00 ± 0.96 | –0.34 ± 0.74 | +0.17 ± 0.93 | 0.00 | 1.00 | 0.00 | 1.00 | 0.00 | 1.00 |
|  | Myopes | +0.26 ± 0.75 | +0.44 ± 0.64 | –0.18 ± 1.00 | +0.46 ± 1.53 |  |  |  |  |  |  |
| *J_45_* | Non-myopes | +0.07 ± 0.99 | –0.17 ± 0.75 | +0.65 ± 0.85 | –0.09 ± 1.93 | 0.61 | 0.44 | 1.49 | 0.22 | 0.99 | 0.40 |
|  | Myopes | –0.08 ± 0.94 | –0.13 ± 0.94 | –0.08 ± 0.81 | +0.12 ± 1.58 |  |  |  |  |  |  |
| *F_L_* | Non-myopes | +22.92 ± 1.89 | +24.82± 1.55 | +27.25 ± 1.66 | +29.78 ± 3.11 | 34.51 | <0.001* | 7.53 | <0.001* | 0.33 | 0.80 |
|  | Myopes | +21.13± 1.54 | +22.42± 1.60 | +25.16 ± 3.07 | +26.90 ± 2.53 |  |  |  |  |  |  |
| Lens shape ratio | Non-myopes | 0.45 ± 0.07 | 0.51 ± 0.07 | 0.59± 0.06 | 0.63 ± 0.08 | 0.16 | 0.69 | 3.51 | 0.02 | 1.12 | 0.33 |
|  | Myopes | 0.44± 0.04 | 0.48± 0.04 | 0.58 ± 0.08 | 0.66± 0.08 |  |  |  |  |  |  |

Figure Supp 3.1. Mean anterior and posterior spherical equivalent lens surface powers *(M)* for the 0, 3, 6, and 9 D accommodation demand for non-myopic and myopic children; error bars represent the standard error of the mean.

*F_L_* increased significantly with accommodation [F_(3, 119)_ = 7.53, p < 0.001] with a significant between group difference [F_(1, 119)_ = 34.51, p < 0.001] such that the power was greater for non-myopes than myopes (Figure Supp 3.2). However, the interaction between accommodation demand and refractive error group was not significant [F_(3, 119)_ = 0.33, p = 0.8], indicating that the change in *F_L_* with accommodation did not differ between groups.

Figure Supp 3.2. Equivalent mean spherical lens power (*F*_L_) at each accommodation demand (0, 3, 6, and 9 D) for non-myopic and myopic groups. Error bars are standard errors of the mean.

The lens shape ratio increased significantly with accommodation [F_(3, 119)_ = 3.51, p = 0.02], but there was no significant between group difference [F_(1, 119)_ = 0.16, p = 0.69] or an accommodation demand by refractive error group interaction [F_(1.90, 41.83)_ = 1.12, p = 0.33].

For the astigmatic refraction vectors, the myopic group had significantly higher anterior lens surface *J_0_* (Figure Supp 3.3) than the non-myopic group [F_(1, 119)_ = 6.9, p = 0.01] across all accommodative demands. The difference (estimated marginal mean ± standard error) was 0.06 ± 0.04 (95% CI: –0.02 to 0.14).

Figure Supp 3.3. Anterior surface *J_0_* power as a function of accommodation demand for non-myopic and myopic children. Error bars are the standard error of the mean.
