# Supplementary material for: Crystalline Lens Shape During Accommodation in Children
Source: Ophthalmic Physiol Opt. 2026 Apr 17;46(3):494–501. doi: 10.1007/s44402-026-00069-5 (PMC13369653; doi:10.1007/s44402-026-00069-5)
Supplement: Supplementary file 4 — Supplementary information [file 44402_2026_69_MOESM4_ESM.docx]

**Supplementary file 1:** Methodology used for optical coherence tomography (OCT) image analysis, outlines the equations used for lens power calculations and repeatability of OCT-based measurements of the lens powers.

**Supplementary file 2:** A **t**able comparing lens parameters between the non-myopic main group (n = 76) and the myopic group (n = 18), with associated statistical outputs corresponding to those presented in the main manuscript. It also provides post-hoc test results between the two refractive groups, as well as an analysis of the relationship between axial length and accommodation-induced changes in lens powers.

**Supplementary file 3:** Comparison between a subset of non-myopic children, age- and sex- matched to the myopic children.
